# Life cycle greenhouse gas emissions of ethanol produced via fermentation of sugars derived from shrub willow (*Salix* ssp.) hot water extraction in the Northeast United States

**DOI:** 10.1186/s13068-021-01900-6

**Published:** 2021-03-01

**Authors:** Obste Therasme, Timothy A. Volk, Mark H. Eisenbies, Thomas E. Amidon, Marie-Odile Fortier

**Affiliations:** 1 Department of Sustainable Resources Management, State University of New York College of Environmental Science and Forestry, Syracuse, NY USA; 2 Department of Paper and Bioprocess Engineering, State University of New York College of Environmental Science and Forestry, Syracuse, NY USA; 3 Department of Civil and Environmental Engineering, University of California, Merced, CA USA

**Keywords:** Life cycle assessment, Willow, Ethanol, Biofuels, Hot water extraction, Fermentation

## Abstract

**Background:**

The amount of carbon dioxide in the atmosphere has been on the rise for more than a century. Bioenergy crops are seen by the Intergovernmental Panel on Climate Change as an essential part of the solution to addressing climate change. To understand the potential impact of shrub willow (*Salix* spp*.*) crop in the northeast United States, effective and transparent life cycle assessment of these systems needs to occur.

**Results:**

Here we show, ethanol produced from the fermentation of sugars from hot water extract of willow grown on cropland can sequester 0.012 ± 0.003 kg CO_2eq_ MJ^−1^ for a supply system incorporating summer harvest and storage. Despite decreases in soil organic carbon when willow is instead grown on grassland, the produced fuel still can provide significant climate benefits compared to gasoline.

**Conclusions:**

Shrub willow converted to ethanol can be a carbon negative source of transportation fuel when the electricity and heat required for the conversion process are generated from renewable biomass. The sequestration of carbon in the belowground portion of the plants is essential for the negative GHG balance for cropland and low GHG emissions in grassland.
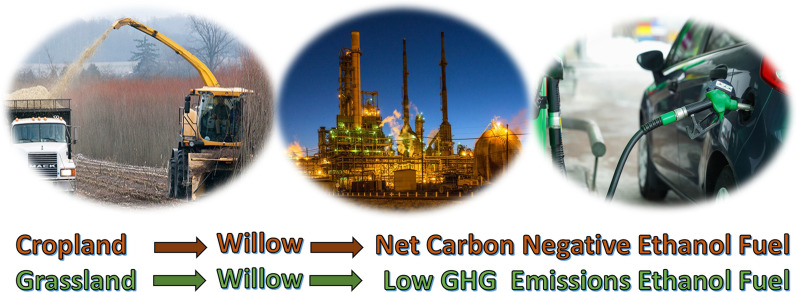

## Background

Global temperatures have been on the rise over the last century and scientists around the world warn that, if no actions are taken to reduce anthropogenic carbon dioxide emissions, global warming could reach 2.8 to 5.5 °C (5 to 10 °F) above pre-industrial level by the end of this century, which would represent serious threats to natural ecosystems and the health and well-being of millions of people [1]. According to the 2018 report of the Intergovernmental Panel on Climate Change (IPPC), total carbon dioxide emissions should be reduced to net zero globally around 2050, to limit global warming at 1.5^0^C above pre-industrial level [1]. In the United States, 29% of the greenhouse gas (GHG) emissions come from the transportation sector [2]. However, a shift to low carbon technologies has started in the transportation sector with the transition from fossil fuels to biofuels (e.g., bioethanol, biodiesel, renewable diesel). Bioethanol in the United States is mainly produced from corn grain and it is the most consumed renewable fuel with 54.5 billion liters used in 2018 [3]. Bioethanol from corn is cost effective and its infrastructure for production, blending with gasoline, and distribution have been deployed nationwide over the last two decades. However, the potential GHG savings from corn ethanol is only in the range of 39–43%, which is still far from being a net zero or net negative GHG emissions transportation fuel [4]. Lignocellulosic ethanol can reach and exceed the 60% GHG reduction mandated by the Renewable Fuel Standard (RFS) program to become a net negative GHG emissions fuel. There is an urgent need to develop new processes, technologies, mechanisms, and energy systems to deliver net zero or net negative carbon fuel sources, i.e., systems with no GHG emissions or with the ability to sequester carbon.

A potential pathway for lignocellulosic ethanol production is via hydrolysis and fermentation to ethanol of carbohydrates extracted via hot water extraction (HWE) [5, 6]. The HWE process is a pretreatment for woody biomass that can yield chemicals and materials such as acetic acid, formic acid, furfural, lignin, and fermentable sugars after multiple separation and purification steps [6–9]. Typically, HWE of woody biomass is performed at elevated temperature in the range of 140 to180^o^C (water is in liquid form) for a period of 30 to 180 min without the use of harsh chemicals, and it results in the solubilization of mostly hemicellulose into the water and the cleavage of acetate groups [10]. Polymeric compounds are converted by auto-hydrolysis reaction into monomeric sugars and oligomers. Recovered sugars from the hydrolysate can be converted into ethanol, butanol, or other high value products via fermentation or other conversion route. The residual HWE biomass has desirable properties such as reduced ash content and increased energy content, making it a valuable feedstock for heat and power cogeneration or pellet production [11].

The development of lignocellulosic biomass to ethanol conversion pathways, high yielding willow (*Salix* spp) cultivars, and commercial scale production in the Northeast US offers new opportunities for a thriving bioeconomy while providing multiple ecosystem services [12, 13]. While there are a few studies on the life cycle GHG emissions of willow production [14–17] and its conversion into ethanol [18–21], they do not incorporate the impacts of direct land use change, the current understanding of the accumulation of willow belowground biomass and harvesting systems, and dry matter losses associated with storage, or incorporate HWE as key step in the conversion process. Budsberg et al. performed a life cycle assessment (LCA) of ethanol production via bioconversion of willow biomass crop feedstock, where steam explosion was used as a pretreatment step, and cellulose and hemicellulose were converted into monomeric sugars by enzymatic hydrolysis prior to the fermentation into ethanol [18]. They reported GHG reductions of up to 120% when comparing ethanol from willow to gasoline, without accounting for the effects of land use change. González-García evaluated the environmental and energy impacts derived from bioethanol production via biochemical conversion of willow established in Sweden [20]. Stephenson assumed simultaneous saccharification and fermentation in a plant that can process 3000 Mg day^−1^ of willow biomass [19]. Stephenson and González-García found GHG savings of 70 to 90% relative to gasoline. An analysis of an HWE-based biorefinery integrated with pulp mills showed a GHG reduction of 80% for the production of concentrated hemicellulose (50% dry solid for five-carbon sugars), 68% for five-carbon sugars that compete with sugar from sugar cane, and a GHG increase of 10% for concentrated hemicellulose (70% dry solid for animal feed) to replace molasses (72% dry solid) from sugar beet [22]. Yet, there is no peer-reviewed assessment of the life cycle impact of bioethanol production from willow feedstocks that incorporates the HWE process.

Soil organic carbon (SOC) is one of the major carbon pools on earth and changes of SOC due to the conversion of current land use to bioenergy is concerning [24]. Field experiments and a developed model of SOC change suggest that cropland conversion to willow biomass results in an overall SOC gain, while the conversion of grassland leads to SOC loss [23]. These changes can increase or decrease the carbon footprint of biofuels from willow biomass and, therefore, need to be included in LCA.

In this study we (a) assess the life cycle GHG emissions associated with the production of ethanol via the fermentation of sugars derived from HWE of willow biomass under different scenarios combining land cover (grassland or cropland converted to willow) and related soil carbon changes, and harvest seasons (summer or winter); (b) identify the variable input parameters to which life cycle GHG emissions are most sensitive; and (c) assess the uncertainty of GHG emissions based on potential values and distributions of variable input parameters. This study integrates models of material capacity and harvester fuel consumption relative to standing biomass from nearly 700 wagon loads of leaf-on and leaf-off willow biomass harvested in the northeast United States [24].

## Results and discussion

### Life cycle GHG emissions

**Fig. 1:**
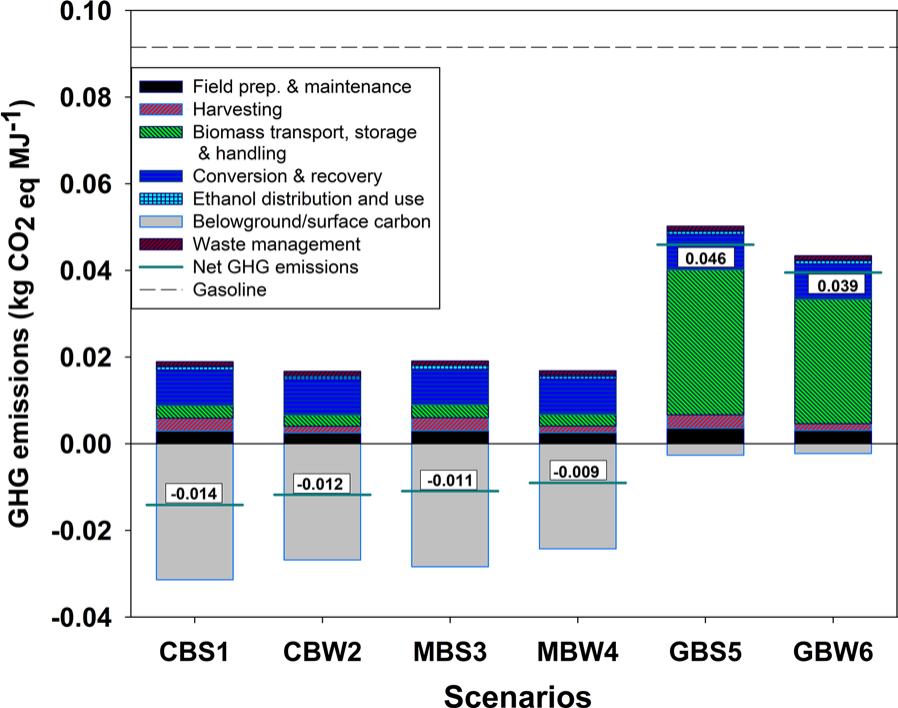
Life cycle GHG emissions by process associated with the production of bioethanol from willow. The number on each bar is the net GHG emissions. Negative values indicate net carbon sequestration from the atmosphere and positive values are net emissions to the atmosphere. For each scenario name, the first letter indicates the land use class before willow was planted (C-cropland, G-grassland, and M-mix of 11.3% G and 88.7% C), the second letter for energy source (B-biomass), and the third letter for harvest season (S-summer and W-winter). The dashed line represents the life cycle GHG emissions of 1 MJ of gasoline

We calculate the cradle-to-grave life cycle GHG emissions on a per unit energy basis (1 MJ) of ethanol produced from willow biomass for different scenarios, considering a biorefinery system that can process 700 Mg (dry) of biomass per day and the utilization of suitable grassland or cropland land to grow willow biomass at commercial scale in northern New York State. For ethanol from willow grown on previous croplands, the life cycle GHG emissions are -0.014 kg CO_2eq_ MJ^−1^ when the biomass is harvested with leaf-on and -0.012 kg CO_2eq_ MJ^−1^ for leaf-off harvest, which indicates that both scenarios (CBS1—cropland biomass summer and CBW2—cropland biomass winter) produce net negative carbon fuels from willow using this HWE process (Fig. 1). Compared to petroleum gasoline, these results translate into GHG emissions reductions of up to 115% (CBS1). For willow grown on previous grasslands, GHG reductions of 50% (GBS5—grassland biomass summer) and 57% (GBW6—grassland biomass winter) are obtained for biomass-based scenarios relative to petroleum gasoline. This study’s results are in the same order of magnitude with previous LCAs of cellulosic ethanol from bioenergy crops, yet with specific differences arising from different assumptions and system designs [18, 19, 25]. For example, a study on ethanol production from wheat reports life cycle GHG emissions of 130 g CO_2eq_ km^−1^, which is equivalent to 0.042 kg CO_2eq_ MJ^−1^ assuming a car efficiency of 0.32 km MJ^−1^ [26]. Another study reports GHG emissions reductions of 77% and 120% for bioethanol produced from willow compared to petroleum gasoline [18], while Stephenson et al. found a 70 to 90% reduction in GHG emissions for ethanol produced from willow by a biochemical conversion process [19]. The results from these studies are more in line with results for the biomass-based energy scenarios (CBS1 and CBW2) as they assumed that lignin, unreacted carbohydrates, and other organic components will be combusted to generate heat and electricity to meet the energy need of the biorefinery facility. On the contrary, other studies that assume carbon-intensive fossil fuel (e.g., coal) as their biorefinery energy source report higher GHG emissions for biofuels than petroleum gasoline [27–29], as it is the case for our system when we assumed that natural gas will be used to meet the energy needs of the biorefinery facility (see Additional file 1).

**Fig. 2:**
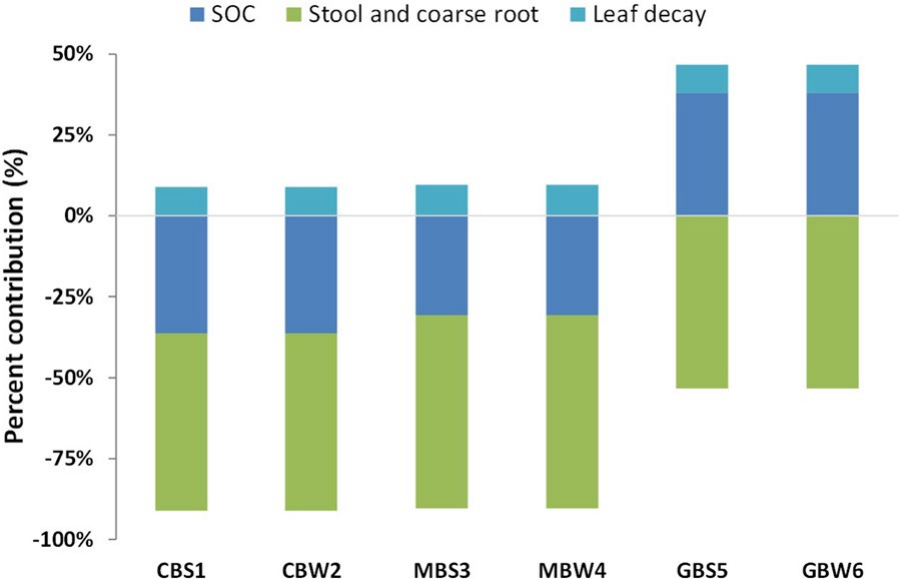
Leaf, roots, and soil organic carbon (SOC) contributions to the belowground/surface GHG carbon emissions

**Fig. 3:**
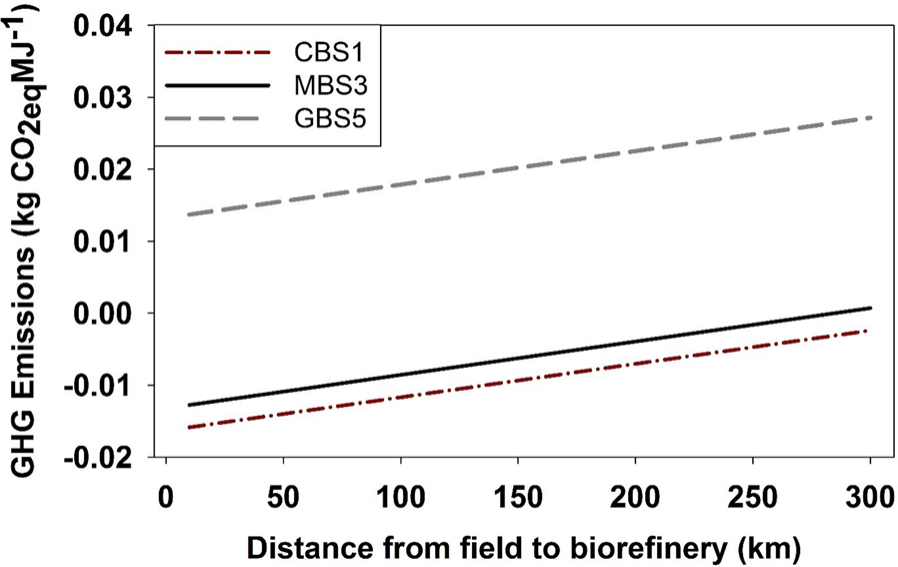
Net GHG emissions as a function of the distance from the fields to the biorefinery. Results shown for the production of bioethanol from HWE of willow biomass grown on cropland (CBS1), grassland (GBS5) or mixed (MBS3). The energy for the biorefinery is supplied by a fraction of the processed biomass residues

The life cycle GHG emissions differences between cropland and grassland scenarios in this study are mostly due to differences in changes in soil organic carbon (SOC) and transportation distance of willow chips from the field to the biorefinery (Figs. 1, 2). At the county level, modeled SOC shows an increase when cropland is converted to willow and it decreases when grassland is converted to willow [23, 25]. The average modeled changes in SOC within the geographic boundary of five counties in New York State (Lewis, Jefferson, Oneida, Saint Lawrence, and Oneida) is 0.29 Mg ha^−1^ year^−1^ (sequestration) in the first 30-cm depth soil for cropland and -0.29 Mg ha^−1^ year^−1^ for grassland over a 22-year period (Table 3 in Methods section). These SOC changes account for 48% of the differences in net life cycle GHG emissions of ethanol between grassland and cropland scenarios. Furthermore, transportation of the biomass to the biorefinery accounts for another 51% of the net life cycle GHG emissions differences. The GHG emissions associated with biomass transportation average to 0.002 kg CO_2eq_ MJ^−1^ for cropland and 0.027 kg CO_2eq_ MJ^−1^ for grassland. The GHG emissions associated with the transportation process are significantly higher for grassland because of a longer transportation distance as a result of fewer suitable grassland parcels than cropland within the geographic boundary of this study. Out of 210,778 ha of suitable parcels identified for willow production within five counties in central and northern New York State, only 11.3% of the areas of suitable parcels are classified as grassland and shrubland [17]. Thus, to collect the same amount of biomass, the transportation distance will be longer for grassland than cropland when we consider the available land distributions in the landscape. To meet the 60% GHG reduction set by the EPA for cellulosic biofuels, several strategies can be used to reduce the transportation distance for grassland scenarios, such as creating smaller HWE processing units across the landscape, blending willow from grassland with willow from cropland (e.g., MBS3, Figs. 1, 3), or maximizing the fraction of suitable grassland parcels to be converted into willow fields. For example, ethanol from willow grown on grassland can provide more than 75% GHG reduction, if the biomass transportation distance from the field to the biorefinery facility is limited to 150 km.

The differences in GHG emissions between summer and winter harvests are mainly driven by higher rates of fuel consumption and dry matter loss (DML) associated with summer harvests. The GHG emissions associated with the processes of site preparation and maintenance, harvesting, transportation, and storage of the biomass are higher for summer than winter harvest and storage for both grassland and cropland. This is the case, because the fuel consumption per unit of harvested willow biomass (L Mg^−1^) during winter harvest is 45% lower than summer harvest and dry matter loss in winter storage piles is 13.8% less than in summer storage piles after 3 months [24, 30]. Thus, the higher dry matter loss and fuel consumption during the summer season contribute higher emissions than during winter by 34% for cropland and 21% for grassland for these processes. Furthermore, the higher dry matter loss means that 14.5% more acres of willow need to be planted, maintained and harvested to meet the biorefinery’s feedstock needs, which also increases the distance that biomass needs to be transported and the amount of carbon in the permanent parts of the willow plant belowground in the root system and aboveground in the stool that is allocated to each MJ of ethanol produced.

### Sensitivity and uncertainty analyses

**Fig. 4:**
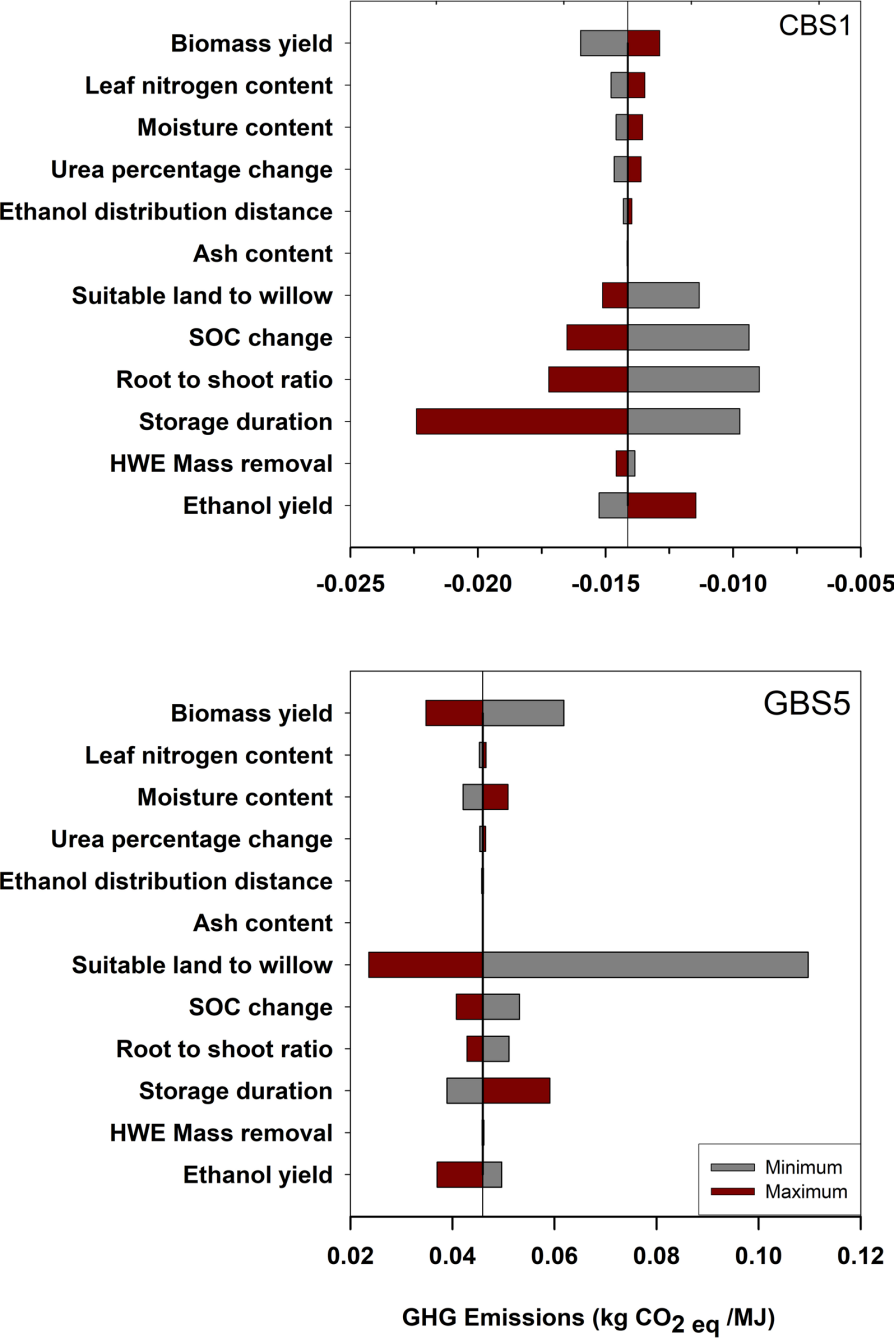
Sensitivity of GHG emissions of bioethanol from willow grown on cropland (CBS1) and grassland (GBS5)

The sensitivity analysis indicates that the variable input parameters that influence net GHG emissions the most are different among all the scenarios (Fig. 4). For scenario CBS1, storage duration, root-to-shoot ratio, and SOC are the most influential variable input parameters, with range of net GHG emissions for bioethanol, in absolute values, of, respectively, 0.013, 0.008, and 0.007 kg CO_2eq_ MJ^−1^, when varying these parameters from their minimum to their maximum values. When these input parameters vary from their baseline to their minimum values, the net life cycle GHG emissions increase by 31 to 36%. In the grassland scenarios, including GBS5, the most sensitive parameter is the proportion of suitable land for willow, because the low concentration of grassland in this region means that a small change in the percentage of grassland available for willow results in disproportionate change in transportation distance from the fields to the biorefinery.

Storage duration has the greatest influence on the net GHG emissions for scenarios that incorporate summer harvest of willow biomass due to the higher rate of dry matter loss in summer storage piles than winter storage piles [30]. Longer storage duration, i.e., higher dry matter loss, results in decreased net GHG emissions compared to the baseline values for all cropland scenarios, but it results in increased net GHG emissions for grassland scenarios. This is the case because of the difference in the direction of the impact associated with SOC changes for cropland and grassland.

Increased biomass yield results in decreased net GHG emissions for grassland but increased emissions for cropland compared to their baseline values. These differences in the direction of the net GHG emissions when varying the biomass yield can be associated with higher carbon sequestration in the underground/surface carbon pool per 1 MJ of ethanol for cropland than grassland. For example, when willow biomass yield on cropland is increased from the baseline value (11.6 Mg ha^−1^) to the maximum value (13.8 Mg ha^−1^), the GHG emissions for CBS1 decrease by 0.001 kg CO_2eq_ MJ^−1^ for harvesting, site preparation, and maintenance, because fewer hectares of land are needed to produce the same amount of biomass. However, these fewer hectares of land mean that there is a smaller increase in soil carbon per MJ and as a result the GHG emissions associated with the underground/surface carbon pool increase by 0.002 kg CO_2eq_ MJ^−1^. The net result is an increase of 0.001 kg CO_2eq_ MJ^−1^. However, for grassland scenarios, these fewer hectares of land translate into smaller soil carbon loss per MJ. These results emphasize the importance of developing a better understanding of changes in soil carbon and belowground biomass over the life cycle of the crop for willow and other bioenergy crops.

The root-to-shoot ratio is another important variable input parameter for many of the scenarios considered under this study as it relates directly to the amount of belowground carbon that is sequestered in shrub willow coarse roots. Increasing the root-to-shoot ratio from the baseline value (0.6) to the maximum value (0.7) increases the carbon sequestration from 0.96 to 1.2 Mg C ha^−1^ year^−1^ on cropland, which translates into a 21% decrease of the baseline net GHG emissions (CBS1) of ethanol from willow. These results suggest that deployment of willow cultivars with a high root-to-shoot ratio can play an important role in mitigating climate change while providing raw materials for biofuels and bioenergy. Nevertheless, additional research is needed to understand the interactions between root-to-shoot ratio and high yielding willow cultivars under the wide range of soil conditions.

For scenarios with natural gas as the fuel source, factors associated with the pretreatment (e.g., HWE mass removal) and conversion processes (e.g., ethanol yield) have the largest impact on GHG emissions. This is to be expected for biorefinery powered by natural gas, because GHG emissions associated with the conversion accounted for over 90% of all emissions in these scenarios. For willow grown on croplands, improvement of ethanol yield or HWE mass removal alone can save, respectively, 0.013 and 0.014 kg CO_2eq_ MJ^−1^, compared to their respective baseline values (Additional file 1).

For grassland scenarios, the fraction of suitable land that is converted to willow production is the most influential input variable parameter to the net GHG emissions. This is due mainly to the high range of this parameter (10% to 100%), the direct relationship between the cumulative suitable area and average travel distance, and the relatively significant contribution of biomass transportation to the net GHG emissions for grassland. Hence, converting 100% of the closest grassland parcels to produce enough biomass to meet the biorefinery demand reduces the transportation distance to 225 km and decreases the net GHG emissions to 0.024 kg CO_2eq_ MJ^−1^ – a 48% reduction compared to the baseline value (GBW5) and a 78% reduction from gasoline.

**Fig. 5:**
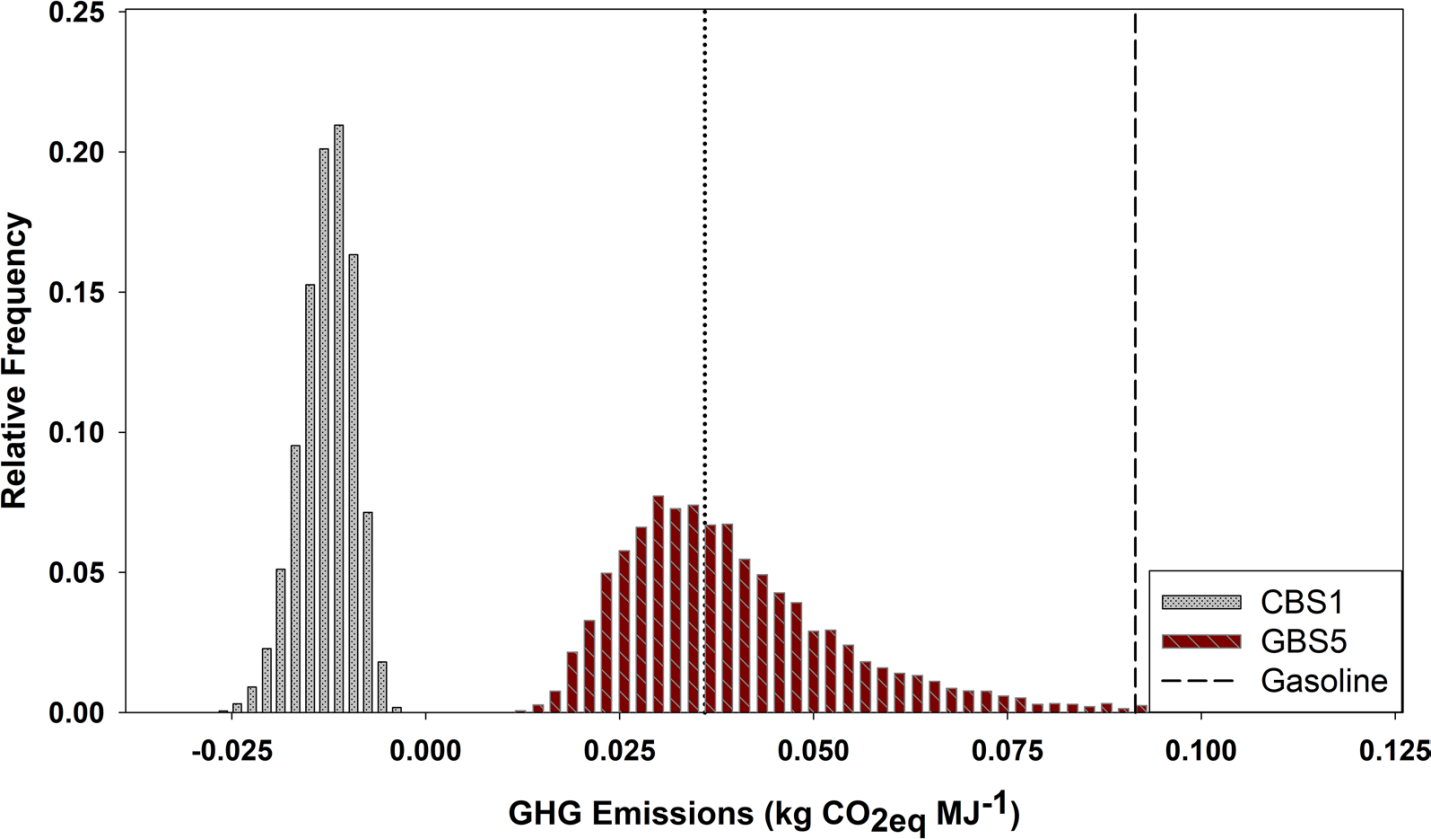
Distribution of GHG emissions of bioethanol from willow grown on cropland (CBS1) and grassland (GBS5). The dotted line represents the 60% GHG emissions reduction required for cellulosic biofuels relative to gasoline (dashed line)

The uncertainty analysis shows that the life cycle GHG emissions values for cropland scenarios are negative with residual biomass as a source of energy (Fig. 5). This indicates that such systems are not only capable of reducing the life cycle GHG emissions compared to petroleum gasoline, but can also act as a carbon sink while producing liquid transportation fuels. Scaling these numbers to the 2.4 to 3.1 million metric tons of willow biomass that could be produced in 2030 in New York State at $80 per ton demonstrates the potential of this system to contribute to a decarbonized economy [31]. Converting this willow biomass to ethanol using the HWE process could produce enough fuel to run (E100) 85,000 to 110,000 cars every year (Additional file 1). With net GHG emissions in the range of -0.008 to -0.019 kg CO_2eq_ MJ^−1^, the production of biofuels from this willow could contribute to a net sequestration of 28 to 86 Gg CO_2eq_ per year and a saving of 345 to 500 Gg CO_2eq_ when accounting for gasoline displacement potential. In addition to these GHG benefits, the expansion of willow across the landscape has the potential to generate a variety of ecosystem services and create 115–150 jobs for every 10,000 ha of willow grown and converted into biofuels [32].

## Conclusions

The production of willow biomass crops and their conversion into biofuels, while using a portion of this biomass as the energy source for the operation can generate a transportation fuel with a negative carbon footprint. This provides an opportunity to meet transportation needs while reducing GHG emissions. This is always the case on cropland, where soil carbon levels increase when planted with perennial willow crops, but when planted on grassland, the biofuel produced is a low carbon (but not net-negative emissions) fuel, because there are projected losses of soil carbon and these areas are widely dispersed with a greater transportation range. The expansion of the current commercial willow biomass system to supply enough feedstock to a commercial scale biorefinery holds significant climate benefits, in term of reduction of GHG emissions from the transportation sector, as well as providing a range of ecosystem services and the creation of new jobs in rural areas. The sensitivity and uncertainty analyses reveal that storage duration, root-to-shoot ratio, biomass yield, and proportion of suitable land converted to willow production are among the most influential variable input parameters. The sequestration of carbon in the belowground portion of the willow plant (roots and stool) and the soils are essential for the negative GHG balance for cropland and low GHG emissions in grassland. There is a need to better understand how these factors change over the life of this crop and how they vary across temporal and spatial scales. This study demonstrates the importance of the energy source on the life cycle GHG emissions of bioethanol, and that ethanol produced from the fermentation of sugars extracted by HWE of willow can be net negative when electricity and heat required for the conversion process are generated from renewable energy sources such as biomass. Further reduction of GHG emissions could be achieved by improving the energy efficiency of HWE and the recovery process for sugars, ethanol, and other co-products.

## Methods

### Goal and scope

**Fig. 6:**
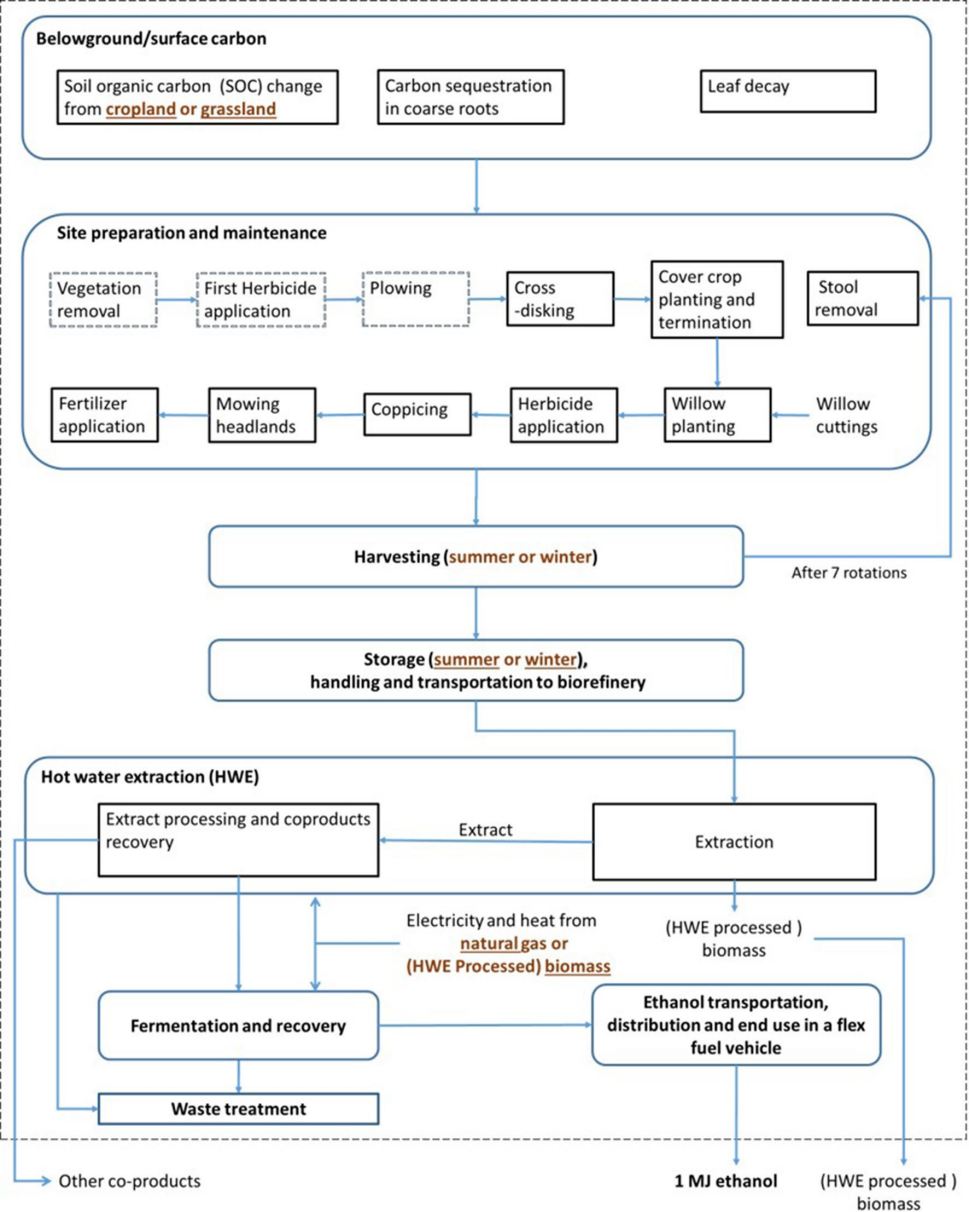
System boundary of bioethanol production by fermentation of sugar from willow hot water extract. Electricity and heat are cogenerated on-site from the combustion of (HWE processed) biomass or natural gas. Site preparation steps of vegetation removal, herbicide applications and plowing (dashed boxes) only apply to grassland. Harvest and storage may occur during the summer or winter seasons. Harvest and fertilizer application occur once during each of the seven 3-year rotations. Headlands around the field are mowed annually. The other site preparation and maintenance steps occur once over the lifetime of the system

This is a cradle-to-grave life cycle assessment (LCA) of bioethanol that is focused on the climate change impact category and performed in accordance to standard methods ISO 14040 and ISO 14044 [33, 34]. The functional unit is 1 megajoule (MJ) of ethanol combusted in a flex fuel vehicle. This functional unit is chosen to facilitate direct comparison of the life cycle GHG emissions of ethanol from willow biomass with ethanol from other biomass feedstocks and petroleum gasoline. The life cycle impacts of materials and chemicals used during willow crop production, HWE extraction, and fermentation of sugars to ethanol are calculated from the Ecoinvent 3 and USLCI databases using the EPA TRACI 2.1 life cycle impact assessment method in SimaPro 8.2 and the GREET 2018 model (see Additional file 1 for a list of the inventories used). The system boundary includes cultivation, harvesting, collection and storage of willow, transportation from the field to the biorefinery facility, hot-water extraction, ethanol fermentation and recovery, as well as the transportation and ethanol fuel combustion in a flex fuel vehicle (Fig. 6). Also, the carbon sequestered underground in root systems, the GHG emissions associated with leaf decomposition, and changes in soil organic carbon (SOC) associated with land use change to grow willow are included inside the system boundary. Whenever it is possible, the impacts of equipment manufacturing and infrastructure are included in the analysis. The impacts of manufacturing are not calculated for equipment used for HWE (reactor, storage tank, pipe) and onsite power generation (wood boiler, turbine, and accessories), and the harvester. These few instances, where the impacts of the infrastructure are omitted, it is expected that it will not have a significant effect on the overall results, as it has been demonstrated in previous LCA studies that biorefinery infrastructures have a relatively small contribution to the life cycle GHG emissions of bioethanol production [35].

This LCA study is based on data collected from a willow biomass system in northern New York State and the model considers seven 3-year rotations of growing and harvesting. It includes multiple scenarios from a combination of previous land uses (grassland and cropland) for willow production, two willow harvest and storage seasons (summer and winter), and two energy sources (natural gas and HWE willow) for the HWE and fermentation processes (Table 1). Previous modeling studies have shown that changes in soil carbon are different when cropland and grassland are converted to willow, and suitable areas of grassland and cropland that can be converted into willow production have been identified for a five-county region in northern New York state [17, 23]. Summer and winter harvests are studied as separate scenarios in this LCA, because they show different harvesting and storage dynamics that affect the mass and energy balance of the system [24, 30]. Heat and power for HWE and fermentation are cogenerated on site either from the combustion of HWE willow biomass or natural gas. Biomass was chosen as an energy source, because most pulp mills or biorefinery systems combust a fraction or all the biomass residues to provide the required heat and power for the plant. Alternatively, natural gas is another fuel source that is attractive due to its low cost, availability, and dispatchability. Sufficient electricity and heat are generated to meet the energy requirement of the biorefinery and any additional electricity is sent to the grid.

**Table 1:** Scenarios from a combination of land use change, harvest season, and energy source

Scenario name	Previous land use	Biorefinery energy source	Harvest season
CBS1	Cropland	Biomass	Summer
CBW2	Cropland	Biomass	Winter
CNS3	Cropland	Natural gas	Summer
CNW4	Cropland	Natural gas	Winter
MBS3	Mixed (11.3% G and 88.7% C)	Biomass	Summer
MBW4	Mixed (11.3% G and 88.7% C)	Biomass	Winter
GBS5	Grassland	Biomass	Summer
GBW6	Grassland	Biomass	Winter
GNS7	Grassland	Natural gas	Summer
GNW8	Grassland	Natural gas	Winter

The proportions on the mixed land use scenario is based on the distribution of cropland and grassland that is needed to meet the annual feedstock demand of a 255,500 Mg biorefinery

Multifunctionality is addressed by process subdivision and mass allocation. During the HWE process, only a fraction of the amount of incoming biomass is extracted, leaving behind a solid residue that can be used as a feedstock for densified biomass fuel (i.e., pellets) or other applications, while the extract results in fermentable sugars and other co-products such as acetic acid, phenolic resin, methanol, furfural, and formic acid [7, 8]. Therefore, the hot water extraction process is divided into two sub-processes: the extraction operation and the processing of the extract. The impact of the extraction sub-process is then split between the extract and the HWE processed willow chips. However, the impact of the extract processing step is not shared with the HWE processed willow chips, but it is split using mass allocation between the fermentable sugars and the other co-products. Furthermore, the impact of the processes associated with willow production and transportation to the biorefinery is allocated by mass among the co-products.

### Data collection

Willow system operation, yield, changes in soil organic carbon from direct land use change (LUC), and transportation distance distribution from field to biorefinery are sourced from a previous analysis with appropriate updates to take into account the differences between scenarios [17]. Advanced Biorefinery Sciences, LLC (ABS, Syracuse, NY) provided detailed data pertaining to energy and mass flows of the HWE process. The most recent research findings on HWE of hardwood and conversion of xylan and glucan into ethanol are applied to this LCA model [5, 6, 36–38]. It is assumed that the distribution distance of ethanol will be 80 km [18]. The GREET model is used to estimate the GHG emissions associated with the combustion of ethanol into a flex-fuel internal combustion vehicle [39].

#### Underground/surface carbon

Carbon can be sequestered in the willow systems for short periods of time in foliage, fine roots, and stem, or long term in coarse roots, aboveground stool, and belowground stools. Long term sequestration in roots and stool is based on root-to-shoot (root-to-standing biomass) ratios determined from trials in central and northern New York [40]. Despite the work done to date showing differences in belowground biomass allocation between high yielding biomass genotypes of willow, the mathematical relationship between biomass yield and underground biomass is still unclear [41]. Therefore, because of the relatively small range of yield values (9.4–13.8 Mg ha^−1^ year^−1^, all biomass values are reported as dry Mg) it is assumed that the root-to-shoot ratio is constant. At the end of the third rotation, the root-to-shoot ratio is 0.6, indicating that, given an aboveground yield of 30 Mg/ha after 3 years of growth, the estimated belowground yield is 18 Mg/ha. The carbon dioxide equivalent absorbed in willow roots is determined by taking into account an average of 460 g of carbon per kilogram of material in the stool and coarse roots, which results in the storage of 1,687 g of carbon dioxide per kilogram [40]. Changes in SOC for 30 cm soil depth resulting from direct land use change of cropland or grassland to willow across five counties in northern NY are informed from county level modeling results generated using a SOC sub model that includes decomposition kinetics and mass balance routines active, slow and passive soil organic matter pools [42]. The modeled results reflect available empirical data for direct land use change for willow [23]. We have used the 30 cm depth values rather than 100 cm, because the marginal land, where willow is grown in the region has soil depths that are restricted by hardpans or perched water tables. The rates of SOC changes are distributed across 22 years. Emissions of nitrous oxide from leaf decomposition are determined based on the International Panel on Climate Change (IPCC) 2006, considering an emissions factor of 1% of the nitrogen released, the amount of leaf litter and leaf nitrogen content, then conversion of the nitrous oxide emissions into carbon dioxide equivalents to facilitate the comparison with other processes [43].

#### Willow production

The comprehensive list of processes in willow biomass production can be found in a previous LCA of willow biomass production which includes site preparation, willow cuttings production and transportation to the field, planting, site management (e.g., application of herbicides and headlands mowing) [17]. By applying filtering criteria on land cover classes, land use types, slope, hydrography, and spatial continuity, suitable parcels to grow willow were identified along with their estimated geometry, the distance of each parcel to the end users, and the biomass yield on these parcels across five counties in central and northern New York [17]. Biomass yield from croplands and grasslands are considered separately in this LCA, with cropland having slightly higher yield than grassland. Relationships (Eqs. 1 and 2) between effective material capacity of a single pass cut and chip harvester, standing biomass yield and harvester efficiency are established (R^2^ = 0.85) for leaf-on (summer) and leaf-off (winter) harvests using data from monitoring of harvesting operations that generated 694 wagon loads of biomass [24, 44]. The harvester fuel consumption is given for leaf-on and leaf-off harvest as a function of standing biomass based on data collected during large scale harvesting operations (Eq. 3). Because of the difficulty in harvesting under wet weather conditions, we only model in this LCA dry weather harvesting operations:

1 $$\begin{aligned} & Willow\; harvester \;material\; capacity{ }_{\left( {winter} \right)} \left( {\frac{Mg}{{hr}}} \right) = 21.2756 + 1.5548 \times Harvester \;Efficiency \\ & \times \;Standing \;Biomass\left( {\frac{Mg}{{ha}}} \right) - 0.0098 \times Harvester \;Effiency \times \left( {Standing\; Biomass \left( {\frac{Mg}{{ha}}} \right)} \right)^{ 2} \\ \end{aligned}$$

2 $$\begin{aligned} & Willow\; harvester\; material\; capacity _{\left( {summer} \right)} \left( {\frac{Mg}{{hr}}} \right) \\ & = 21.2756 - 14.4187 Harvester\; Efficiency - 0.7223 Standing\; Biomass\left( {\frac{Mg}{{ha}}} \right) \\ & + 0.0051 \left( {Standing Biomass\left( {\frac{Mg}{{ha}}} \right)} \right)^2 + 1.5548 Harvester \; Efficiency \\ & \times Standing \;Biomass\left( {\frac{Mg}{{ha}}} \right) - 0.0098 Harvester\; Efficiency \times \left( {Standing \;Biomass \left( {\frac{Mg}{{ha}}} \right)} \right) ^2 \\ \end{aligned}$$

3 $$\begin{aligned} & {\text{Willow}}\;{\text{harvester}}\;{\text{fuel}}\;{\text{consumption}} \left( {\frac{{L}}{{hr}}} \right) = 1.466 \times {\text{Material}}\;{\text{capacity}} \left( {\frac{{Mg}}{{hr}}} \right) \times \left( {\frac{\text{A}}{\text{Standing biomass (Mg/ha)}}} + \text{B} \right ) \end{aligned}$$

where the coefficient A is equal to 64.1803 and 20.8959, respectively, for leaf-on (summer) harvest and leaf-off (winter) harvest and the coefficient B is equal to 1.1559 and 0.8582, respectively, for leaf-on (summer) harvest and leaf-off (winter) harvest.

Furthermore, it is assumed that harvested willow chips will be stored in outdoor piles for a period ranging from zero to 6 months with an average storage period of 3 months as the baseline value. Changes in dry matter loss (DML) and quality changes during the summer and winter storage are from recent storage trial studies of willow [30, 45]. After 3 months in storage, the average DML is estimated at 18.9% for summer storage and 5.2% for winter storage (Eq. 4):

4 $$DML \left( \% \right) = - 2.3283 + A \times Period \left( {days} \right),$$

where “DML” is the percentage of dry matter loss during storage in summer and winter storage piles and “Period” is the number of days of storage. The coefficient A is 0.2369 and 0.0836, respectively, for summer storage pile and winter storage pile. The minimum DML value equals zero.

*Feedstock Transportation*: Biomass transportation covers moving the willow biomass from the edge of the field to the biorefinery gate. The transportation distance of the biomass is scaled to the annual capacity of the biorefinery (700 Mg day^−1^ or 255,500 Mg dry) and weighed by the biomass yield and area of each parcel (see Additional file 1). The total area of suitable grassland is significantly less than cropland within the geographic boundary of the study [17]. Thus, the average transportation distance (Eq. 5) to supply the same amount of biomass is significantly higher for grassland than cropland. For example, to meet an annual demand of 255,500 Mg of willow chips, the average transportation distance to the biorefinery would be 26 km (52 km round trip) for cropland, assuming that all the identified parcels were to be converted into willow fields. For grassland, it would be an order of magnitude higher. It is not guaranteed that all the suitable grassland or cropland will be turned into willow, considering the potential role of the landowner in such a decision. Therefore, the transportation distance varies with the fraction of suitable lands that are devoted to willow to meet the needs of the biorefinery. An average of 30% of suitable land is assumed to be converted into willow. This LCA model captures the relationship between transportation distance and biomass yield, fraction of suitable land converted to willow, and dry matter loss during biomass storage. When dry matter loss increases, more land area will have to be converted to willow to meet the needs of the biorefinery, and the transportation distance from the field to the biorefinery increases as well to provide the required biomass. The lowest transportation distance will be obtained when the highest yield is combined with the highest fraction of land devoted to willow production for a given biomass input:

5 $$Average Distance \left( {km} \right) = A + B \times \left( {1 + headland} \right) \times \left( {\frac{100}{{100 - DML}}} \right) \times \left( {\frac{100}{{SL}}} \right) \times \frac{{Annual Biorefinery Capacity\left( {\frac{Mg}{{yr}}} \right)}}{{Biomass Yield \left( {\frac{Mg}{{ha.yr}}} \right)}} ,$$

where the coefficient A is equal to 17.027 and 17.578, respectively, for cropland and grassland and the coefficient B is equal to 0.0003 and 0.0063, respectively, for cropland and grassland. The headland represents the fraction (about 0.1) of the total acreage of a field that is left unplanted for equipment access. SL is the percentage of suitable land that is expected to be converted to willow production.

#### Conversion and separation

The biorefinery system is designed to process the equivalent of 700 Mg (oven dry) woodchips per day and produce 50,130 L of ethanol. Chipped willow biomass is transported to the gate of the biorefinery, and then transferred to the reactor, where the woodchips are mixed with water (water to wood ratio of 4:1) and heated at 160^0^C for 2 h. The stack gas is used for pre-drying of the processed wood chips to reduce its moisture content to 42% (wet basis). The extract is separated by filtration from the solids and then submitted to multiple separation steps including membrane separation and centrifugation, and acid hydrolysis of oligomers. The extract yields acetic acid, methanol, formic acid, lignin, furfural, and fermentable sugars (Table 2). Sulfuric acid is used to foster the decomposition of sugar polymers into fermentable monomeric sugars. Sodium hydroxide is used to neutralize the acid waste. Then, the fermentable sugars mixture is transferred to the fermenter, where it can be converted into ethanol using specially selected strains of yeasts [5]. The maximum theoretical yield for glucose and xylose fermentation is 51.1% on a mass basis under anaerobic conditions. Under aerobic conditions, partial oxidation reaction reduces the theoretical yield to 46.03% [6]. The fermentation of HWE hydrolysate by an adapted strain of *Pichia stipilis* produces 0.406 g ethanol per gram of sugar under microaerobic conditions (~ 2% oxygen after 12 h) with a residence time of 39 h [5]. The ethanol recovery process, however, is based on the 2011 NREL (National Renewable Energy Laboratory) updated model for lignocellulosic biomass to ethanol [46]. This model was chosen, because it includes research progress in optimization of products recovery and incorporates realistic configurations for critical equipment. The resulting mixture of ethanol and water will be separated by distillation to 92.5% ethanol and dehydrated by vapor-phase molecular sieve adsorption to 99.5%.

**Table 2:** Consumption and production of major chemicals and products during the willow harvesting, hot water extraction, fermentation and ethanol recovery

	Unit	Previous land use	
		Cropland	Grassland
Harvesting			
Harvester fuel consumption (leaf-on)	L/Mg	5.7	6.0
Harvester fuel consumption (leaf-off)	L/Mg	3.1	3.2
Harvested biomass (leaf on)	10^3^ Mg/year	315	315
Harvested biomass (leaf off)	10^3^ Mg/year	269	269

		Energy source	
		Biomass	Natural gas
HWE Input			
Wood chips	Mg/day	700	700
Sulfuric acid	Mg/day	3.28	3.28
Hydrochloric acid	Mg/day	0.41	0.41
Calcium hydroxide	Mg/day	3.06	3.06
Sodium hydroxide	Mg/day	0.04	0.04
Formic acid	Mg/day	2.07	2.07
Flocculant	Mg/day	0.15	0.15
Membrane (8 × 40″)	unit/year	1527	1527
Water	10^3^ Mg/day	4.2	4.2
Energy requirement (extraction)	GJ/day	501	506
Energy requirement (extract processing)	GJ/day	750	758
HWE Output			
HWE processed wood chips	Mg/day	393^a^	543^a^
Sugars	Mg/day	97.4	97.4
Acetic acid	Mg/day	17.3	17.3
Phenolic resins	Mg/day	28.0	28.0
Methanol	Mg/day	5.04	5.04
Furfural	Mg/day	3.61	3.61
Fermentation and recovery Input			
Energy requirement (fermentation)	GJ/day	23.0	23.0
Energy requirement (distillation)	GJ/day	304	304
Initial cell concentration (10% v)	g/g sugar	0.044	0.044
Fermentation and recovery Output			
Ethanol	Mg/day	39.6	39.6
Waste treatment Output			
Waste water	Mg/day	1760	1760
Ash	Mg/day	1.86	0.00
Excess electricity			
Electricity	GJ/day	134	146

^a^Fewer HWE chips leave the system boundary under the biomass scenario than natural gas, because a fraction of the HWE chips is combusted in a combined heat and power (CHP) system to meet the heat and electricity requirement of the biorefinery

#### Sensitivity and uncertainty analyses

This study includes sensitivity and uncertainty analyses using Python 2.7 for each scenario to capture the variability of the life cycle GHG emissions associated with the production of ethanol from willow under a range of potential conditions. The sensitivity analysis is performed by varying an input parameter from its baseline value to its minimum and maximum values while keeping all other variable parameters at their baseline values (Table 3). The Monte Carlo analysis is conducted by selecting random values from an assigned probability distribution for each variable input parameter to quantify the uncertainty of the GHG emissions. The number of generated scenarios with different combinations of input values for the Monte Carlos analysis is 10,000. For parameters with fewer than 25 data points and unknown probability distributions, we assume triangular probability distributions.

**Table 3:** Variable input parameters used in the sensitivity and uncertainty analyses

Variable parameters	Unit		Minimum		Baseline		Maximum		Sources
Ash content of HWE willow	%		0.2		1.2		2.8		[9]
Willow biomass yield^a^									
Cropland	Mg ha^−1^ year^−1^		9.4		11.6		13.8		[17]
Grassland	Mg ha^−1^ year^−1^		8.2		10.7		13.6		
Ethanol distribution distance	km		60		80		100		[18]
Ethanol yield	g/g		0.37		0.41		0.51		[5]
HWE Mass removal	%		19.9		22.4		26.6		[9]
Leaf nitrogen content	%		1.88		2.32		2.78		[17, 40]
Moisture content^a^	% (wet basis)		37		44		51		[47]
Root to shoot ratio	_		0.46		0.61		0.7		[40]
SOC change									
Cropland	Mg C ha^−1^ year^−1^	0.19		0.29		0.34		[17, 23, 25]	
Grassland	Mg C ha^−1^ year^−1^	− 0.43		− 0.29		− 0.19			
Storage duration	Month	0		3		6		Estimation	
Percent of suitable land used	%	10		30		100		Estimation	
Urea	kg N ha^−1^	80		100		120		[48]	

^a^Biomass yield best fits a logistic probability distribution type and moisture data fits a normal probability distribution. The probability distributions of the other parameters are unknown because of insufficient numbers of data points to run a best fit analysis. We assumed a triangular probability distribution for these variable parameters. The baseline values of biomass yield are the median values across a range of field within the geographic boundary of this analysis, and the minimum and maximum values correspond to 95% confidence interval of these yield data. A minimum of 10% of the total amount of suitable land within the geographic boundary of this analysis is required to supply 700 Mg of willow chips to the biorefinery daily

## Supplementary Information

**Additional file 1: Table S1.1.** Sensitivity analysis results (kg CO_2eq_ MJ^-1^) for multiple scenarios,** Table S1.2.** Estimation of the potential net GHG sequestration and number of car equivalent emissions,** Table S2.1:** List of life cycle inventories used from databases and peer reviewed literature,** S2.2:** Linear equation linking the cumulative area of suitable parcels for willow production and weighed distance by the yield and area of each parcel for cropland, and** S2.3:** Linear equationlinking the cumulative area of suitable parcels for willow production and weighed distance by the yield and area of each parcel for grassland.

## Data Availability

The dataset supporting the conclusions of this article is included within the article and its additional file.

## Abbreviations

CBS1: Cropland, biomass and summer
CBW2: Cropland, biomass and winter
CNS3: Cropland, natural gas and summer
CNW4: Cropland, natural gas and winter
MBS3: Mixed cropland and grassland, biomass and summer
MBW4: Mixed cropland and grassland, biomass and winter
GBS5: Grassland, biomass and summer
GBW6: Grassland, biomass and winter
GNS7: Grassland, natural gas and summer
GNW8: Grassland, natural gas and winter
GHG: Greenhouse gas
HWE: Hot water extraction
LCA: Life cycle assessment
SOC: Soil organic carbon
