# Supplementary material for: Life cycle greenhouse gas emissions of ethanol produced via fermentation of sugars derived from shrub willow (Salix ssp.) hot water extraction in the Northeast United States
Source: Biotechnol Biofuels. 2021 Mar 1;14:52. doi: 10.1186/s13068-021-01900-6 (PMC7923501; doi:10.1186/s13068-021-01900-6)
Supplement: Supplementary file 1 — Additional file 1: Table S1.1. Sensitivity analysis results (kg CO2eq MJ-1) for multiple scenarios, Table S1.2. Estimation of the potential net GHG sequestration and number of car equivalent emissions, Table S2.1: List of life cycle inventories used from databases and peer reviewed literature, S2.2: Linear equation linking the cumulative area of suitable parcels for willow production and weighed distance by the yield and area of each parcel for cropland, and S2.3: Linear equationlinking the cumulative area of suitable parcels for willow production and weighed distance by the yield and area of each parcel for grassland. [file 13068_2021_1900_MOESM1_ESM.docx]

**Life Cycle Greenhouse Gas Emissions of Ethanol Produced via Fermentation of Sugars Derived from Shrub Willow (Salix ssp.) Hot Water Extraction in the Northeast United States**

**(Supplementary Information)**

Obste Therasme^1^, Timothy A. Volk^1^, Mark H. Eisenbies^1^, Thomas E. Amidon^2^, and Marie-Odile Fortier^3*^

^1^ Department of Sustainable Resources Management, State University of New York College of Environmental Science and Forestry, Syracuse, NY, United States of America

^2^ Department of Paper and Bioprocess Engineering, State University of New York College of Environmental Science and Forestry, Syracuse, NY, United States of America

^3^ Department of Civil and Environmental Engineering, University of California, Merced, CA, United States of America

^*^Corresponding Author’s email: mfortier2@ucmerced.edu

S1.1. Sensitivity analysis results (kg CO_2eq_ MJ^-1^) for multiple scenarios to produce ethanol from willow hot water extracted. The values shown are the life cycle GHG emissions when the variable parameters are changed to their minimum (min) or maximum (max) values from their baseline values.

| Variable parameters* | CBS1 | | | CBW2 | | | CNS3 | | | CNW4 | | | GBS5 | | | GBW6 | | | GNS7 | | | GNW8 | | |
| --- | --- | --- | --- | --- | --- | --- | --- | --- | --- | --- | --- | --- | --- | --- | --- | --- | --- | --- | --- | --- | --- | --- | --- | --- |
|  | Min | Baseline | Max | Min | Baseline | Max | Min | Baseline | Max | Min | Baseline | Max | Min | Baseline | Max | Min | Baseline | Max | Min | Baseline | Max | Min | Baseline | Max |
| Ash content | -0.014 | -0.014 | -0.014 | -0.012 | -0.012 | -0.012 | 0.076 | 0.076 | 0.076 | 0.078 | 0.078 | 0.078 | 0.046 | 0.046 | 0.046 | 0.039 | 0.039 | 0.040 | 0.123 | 0.123 | 0.123 | 0.118 | 0.118 | 0.118 |
| Biomass yield | -0.016 | -0.014 | -0.013 | -0.014 | -0.012 | -0.011 | 0.075 | 0.076 | 0.077 | 0.077 | 0.078 | 0.079 | 0.062 | 0.046 | 0.035 | 0.053 | 0.039 | 0.030 | 0.136 | 0.123 | 0.115 | 0.129 | 0.118 | 0.111 |
| Ethanol distribution distance | -0.014 | -0.014 | -0.014 | -0.012 | -0.012 | -0.012 | 0.076 | 0.076 | 0.076 | 0.078 | 0.078 | 0.078 | 0.046 | 0.046 | 0.046 | 0.039 | 0.039 | 0.040 | 0.123 | 0.123 | 0.124 | 0.118 | 0.118 | 0.119 |
| Ethanol yield | -0.015 | -0.014 | -0.011 | -0.013 | -0.012 | -0.010 | 0.080 | 0.076 | 0.067 | 0.082 | 0.078 | 0.069 | 0.050 | 0.046 | 0.037 | 0.043 | 0.039 | 0.032 | 0.131 | 0.123 | 0.105 | 0.126 | 0.118 | 0.101 |
| HWE Mass removal | -0.014 | -0.014 | -0.015 | -0.012 | -0.012 | -0.012 | 0.082 | 0.076 | 0.069 | 0.084 | 0.078 | 0.070 | 0.046 | 0.046 | 0.046 | 0.039 | 0.039 | 0.040 | 0.130 | 0.123 | 0.116 | 0.125 | 0.118 | 0.111 |
| Leaf nitrogen content | -0.015 | -0.014 | -0.013 | -0.012 | -0.012 | -0.011 | 0.076 | 0.076 | 0.077 | 0.078 | 0.078 | 0.079 | 0.045 | 0.046 | 0.047 | 0.039 | 0.039 | 0.040 | 0.123 | 0.123 | 0.124 | 0.118 | 0.118 | 0.119 |
| Moisture content | -0.015 | -0.014 | -0.014 | -0.012 | -0.012 | -0.011 | 0.076 | 0.076 | 0.077 | 0.078 | 0.078 | 0.079 | 0.042 | 0.046 | 0.051 | 0.036 | 0.039 | 0.044 | 0.120 | 0.123 | 0.127 | 0.116 | 0.118 | 0.122 |
| Root to shoot ratio | -0.009 | -0.014 | -0.017 | -0.007 | -0.012 | -0.014 | 0.080 | 0.076 | 0.074 | 0.082 | 0.078 | 0.076 | 0.051 | 0.046 | 0.043 | 0.044 | 0.039 | 0.037 | 0.128 | 0.123 | 0.121 | 0.122 | 0.118 | 0.116 |
| SOC change | -0.009 | -0.014 | -0.017 | -0.008 | -0.012 | -0.014 | 0.080 | 0.076 | 0.074 | 0.081 | 0.078 | 0.077 | 0.053 | 0.046 | 0.041 | 0.046 | 0.039 | 0.035 | 0.129 | 0.123 | 0.119 | 0.123 | 0.118 | 0.115 |
| Storage duration | -0.010 | -0.014 | -0.022 | -0.011 | -0.012 | -0.014 | 0.080 | 0.076 | 0.070 | 0.079 | 0.078 | 0.077 | 0.039 | 0.046 | 0.059 | 0.038 | 0.039 | 0.042 | 0.118 | 0.123 | 0.134 | 0.117 | 0.118 | 0.120 |
| Percent suitable land | -0.011 | -0.014 | -0.015 | -0.009 | -0.012 | -0.013 | 0.079 | 0.076 | 0.076 | 0.080 | 0.078 | 0.078 | 0.110 | 0.046 | 0.024 | 0.094 | 0.039 | 0.020 | 0.174 | 0.123 | 0.106 | 0.161 | 0.118 | 0.103 |
| Urea | -0.015 | -0.014 | -0.014 | -0.012 | -0.012 | -0.011 | 0.076 | 0.076 | 0.077 | 0.078 | 0.078 | 0.079 | 0.045 | 0.046 | 0.047 | 0.039 | 0.039 | 0.040 | 0.123 | 0.123 | 0.124 | 0.118 | 0.118 | 0.119 |

* The ranges of the variable input parameters are reported in Table 3. The definition of each scenario is included in Table 1.

S1.2. Estimation of the potential net GHG sequestration and number of car equivalent emissions if producing ethanol fuel from willow biomass grown on cropland (CBS1) considering the uncertainties of the net GHG emissions.

| High yield scenario (BTS^a^) applied to CBS1 Scenario (New York, 2030) | | | | |
| --- | --- | --- | --- | --- |
|  | | P=0.05^b^ less than  -0.019 kg CO_2eq_ MJ^-1^ | Median Value (P=0.50) | P=0.95 less than  -0.008 kg CO_2eq_ MJ^-1^ |
| Total land area ^a^ | acres | 1.11E+05 | 1.11E+05 | 1.11E+05 |
| Biomass Production ^a^ | Mg yr ^-1^ | 2.42E+06 | 2.42E+06 | 2.42E+06 |
| Net GHG Sequestration | Gg yr ^-1^ | 4.19E+01 | 6.63E+01 | -2.79E+01 |
| Net GHG saving (incl. gasoline displacement) | Gg yr ^-1^ | 3.60E+02 | 3.84E+02 | 3.46E+02 |
| Number of cars equivalent | cars | 1.03E+05 | 9.63E+04 | 9.26E+04 |
| Baseline Scenario (BTS) applied to CBS1 Scenario (New York, 2030) | | | | |
|  | | P=0.05 less than -0.019 kg CO2/MJ | Median (P=0.50) | P=0.95 less than 0.008 |
| Total land area ^a^ | acres | 1.53E+05 | 1.53E+05 | 1.53E+05 |
| Biomass Production ^a^ | Mg yr ^-1^ | 3.14E+06 | 3.14E+06 | 3.14E+06 |
| Net GHG Sequestration | Gg yr ^-1^ | 5.43E+01 | 8.60E+01 | 3.62E+01 |
| Net GHG saving (gasoline displacement) | Gg yr ^-1^ | 4.66E+02 | 4.98E+02 | 4.48E+02 |
| Number of cars equivalent | cars | 1.33E+05 | 1.25E+05 | 1.20E+05 |

^a^ BTS: Billion-ton Study. Total area of land that can be used to produce willow biomass in NY State in 2030 at $80 per ton and estimated production capacity numbers are sourced from the billion-ton study^1^. The fuel efficiency of a passenger car is 16 km/l and an average person drives about 19,200 km per year^2^.

^b^ GHG emissions of ethanol from the uncertainty analysis. There is a 5% probability that the emissions are less than -0.019 kg CO_2eq_ MJ^-1^ and 95% probability that is less than -0.008 kg CO_2eq_ MJ^-1^.

1. Langholtz, M. H., Stokes, B. J. & Eaton, L. M. 2016 Billion-Ton Report: Advancing Domestic Resources for a Thriving Bioeconomy. http://www.osti.gov/servlets/purl/1271651/ (2016) doi:10.2172/1271651.
2. Bureau of Transportation Statistics (https://www.bts.gov/content/average-fuel-efficiency-us-passenger-cars-and-light-trucks)

S2.1: List of life cycle inventories used from databases and peer reviewed literature.

| Inventory | Source |
| --- | --- |
| Bale loading | Ecoinvent 3 |
| Calcium hydroxide | Ecoinvent 3 |
| Diesel | USLCI |
| Gasoline | GREET 2018 model |
| Disking | Ecoinvent 3 |
| Electricity NY UP | USLCI |
| Ethanol combustion | GREET model |
| Ethanol fermentation plant construction | Ecoinvent 3 |
| Field sprayer | Ecoinvent 3 |
| Herbicide | Ecoinvent 3 |
| Landfill of ash from paper mill | Ecoinvent 3 |
| Lime application | Ecoinvent 3 |
| Lubricant oil | Ecoinvent 3 |
| Membrane module | Tangsubkul et al. 2006 |
| Mow rake | Ecoinvent 3 |
| Natural gas combusted in industrial equipment | USLCI |
| Planting | Ecoinvent 3 |
| Plowing | Ecoinvent 3 |
| Roller crimper | Ecoinvent 3 |
| Sodium hydroxide | Ecoinvent 3 |
| Sulfuric acid | Ecoinvent 3 |
| Tractor | Ecoinvent 3 |
| Transportation truck | USLCI |
| Urea | Ecoinvent 3 |
| Wagon/loader | Ecoinvent 3 |
| Wastewater treatment | Ecoinvent 3 |
| Water | Ecoinvent 3 |
| Willow cuttings | Caputo et al. 2014 |
| Yeast production | Ecoinvent 3 |

S2.2: Linear equation linking the cumulative area of suitable parcels for willow production and weighed distance by the yield and area of each parcel for cropland type in central and northern New York. The area and transportation distance of each parcel to the end user were determined from a spatial analysis using GIS tools (from Yang, 2017).

Cropland

| Cumulative area (ha) | Weighed Distance (km) |
| --- | --- |
| 11.07 | 6.45786 |
| 21.33 | 6.47924 |
| 32.76 | 6.67494 |
| 40.95 | 6.80401 |
| 64.17 | 6.99495 |
| 88.11 | 7.12296 |
| 97.65 | 7.16549 |
| 140.04 | 7.35956 |
| 161.82 | 7.43402 |
| 179.46 | 7.4983 |
| 208.71 | 7.58941 |
| 220.5 | 7.62055 |
| 237.51 | 7.66033 |
| 247.32 | 7.68961 |
| 256.05 | 7.71557 |
| 288.81 | 7.7999 |
| 342.27 | 7.92474 |
| 377.1 | 8.0043 |
| 410.49 | 8.06863 |
| 442.71 | 8.12866 |
| 456.93 | 8.15325 |
| 468.72 | 8.17317 |
| 477.27 | 8.19043 |
| 491.4 | 8.21808 |
| 503.55 | 8.24192 |
| 524.25 | 8.28448 |
| 538.38 | 8.31456 |
| 571.5 | 8.38055 |
| 585.63 | 8.40693 |
| 612.09 | 8.45365 |
| 623.97 | 8.47437 |
| 634.77 | 8.49291 |
| 647.01 | 8.51433 |
| 678.15 | 8.56656 |
| 711.18 | 8.61936 |
| 723.42 | 8.6382 |
| 735.66 | 8.65785 |
| 755.46 | 8.68847 |
| 772.92 | 8.71428 |
| 788.58 | 8.73754 |
| 803.25 | 8.76073 |
| 830.16 | 8.80121 |
| 838.89 | 8.81383 |
| 876.42 | 8.86729 |
| 899.01 | 8.89836 |
| 933.21 | 8.94326 |
| 945.09 | 8.96065 |
| 953.37 | 8.97256 |
| 990.27 | 9.02398 |
| 1002.15 | 9.03984 |
| 1014.66 | 9.05692 |
| 1033.65 | 9.08239 |
| 1064.34 | 9.12294 |
| 1082.88 | 9.14653 |
| 1097.46 | 9.16466 |
| 1160.73 | 9.23823 |
| 1201.59 | 9.28424 |
| 1243.71 | 9.32875 |
| 1278.54 | 9.36579 |
| 1287.99 | 9.376 |
| 1301.85 | 9.3919 |
| 1327.14 | 9.42128 |
| 1383.39 | 9.48579 |
| 1404.36 | 9.50855 |
| 1427.49 | 9.53471 |
| 1440.9 | 9.54962 |
| 1452.06 | 9.56222 |
| 1465.38 | 9.57725 |
| 1488.6 | 9.60288 |
| 1500.39 | 9.61598 |
| 1529.37 | 9.64801 |
| 1545.3 | 9.6661 |
| 1565.82 | 9.68972 |
| 1589.76 | 9.71819 |
| 1600.11 | 9.7304 |
| 1621.44 | 9.75678 |
| 1655.46 | 9.80006 |
| 1668.96 | 9.81702 |
| 1678.77 | 9.82942 |
| 1717.47 | 9.87745 |
| 1733.4 | 9.89731 |
| 1747.17 | 9.91428 |
| 1755.72 | 9.9247 |
| 1769.49 | 9.94143 |
| 1785.06 | 9.96014 |
| 1797.66 | 9.97511 |
| 1867.23 | 10.0564 |
| 1877.49 | 10.0684 |
| 1885.5 | 10.0776 |
| 1894.5 | 10.0879 |
| 1906.65 | 10.1023 |
| 1931.31 | 10.1312 |
| 1973.52 | 10.1798 |
| 1982.43 | 10.1901 |
| 1997.46 | 10.2075 |
| 2013.75 | 10.2262 |
| 2028.24 | 10.2427 |
| 2042.64 | 10.2592 |
| 2051.28 | 10.269 |
| 2081.34 | 10.3027 |
| 2092.95 | 10.3155 |
| 2110.23 | 10.3345 |
| 2121.66 | 10.347 |
| 2132.73 | 10.359 |
| 2142.63 | 10.3699 |
| 2169.36 | 10.3988 |
| 2184.93 | 10.4156 |
| 2196.99 | 10.4285 |
| 2205.45 | 10.4375 |
| 2220.3 | 10.4534 |
| 2234.7 | 10.4688 |
| 2243.61 | 10.4784 |
| 2268.9 | 10.5057 |
| 2284.02 | 10.5218 |
| 2292.03 | 10.5304 |
| 2311.92 | 10.5515 |
| 2322.27 | 10.5623 |
| 2351.79 | 10.5928 |
| 2366.91 | 10.6082 |
| 2376.45 | 10.6179 |
| 2389.32 | 10.6311 |
| 2429.82 | 10.674 |
| 2452.41 | 10.6978 |
| 2473.74 | 10.7199 |
| 2482.83 | 10.7294 |
| 2510.82 | 10.7585 |
| 2521.62 | 10.7696 |
| 2542.77 | 10.7917 |
| 2551.95 | 10.8013 |
| 2576.88 | 10.8273 |
| 2591.01 | 10.8419 |
| 2611.17 | 10.8632 |
| 2624.85 | 10.8776 |
| 2637.63 | 10.891 |
| 2649.6 | 10.9035 |
| 2663.82 | 10.9182 |
| 2673.45 | 10.9282 |
| 2684.52 | 10.9397 |
| 2695.77 | 10.9514 |
| 2718.54 | 10.9751 |
| 2752.74 | 11.0107 |
| 2763 | 11.0214 |
| 2781.36 | 11.0407 |
| 2804.67 | 11.0649 |
| 2815.92 | 11.0766 |
| 2832.75 | 11.0939 |
| 2852.82 | 11.1145 |
| 2867.13 | 11.129 |
| 2879.82 | 11.1419 |
| 2897.46 | 11.1597 |
| 2905.83 | 11.1682 |
| 2924.46 | 11.1869 |
| 2947.95 | 11.2104 |
| 2965.86 | 11.2283 |
| 2973.87 | 11.2363 |
| 2985.03 | 11.2475 |
| 2997.27 | 11.2597 |
| 3012.75 | 11.2752 |
| 3029.22 | 11.2918 |
| 3037.77 | 11.3003 |
| 3051.54 | 11.3142 |
| 3107.16 | 11.3701 |
| 3131.73 | 11.3942 |
| 3143.97 | 11.4061 |
| 3170.43 | 11.4315 |
| 3178.98 | 11.4398 |
| 3195.99 | 11.4563 |
| 3204.45 | 11.4647 |
| 3213.72 | 11.474 |
| 3223.62 | 11.4839 |
| 3236.85 | 11.4972 |
| 3245.76 | 11.5062 |
| 3265.65 | 11.5263 |
| 3292.11 | 11.5532 |
| 3302.28 | 11.5636 |
| 3311.28 | 11.5728 |
| 3329.01 | 11.5908 |
| 3337.65 | 11.5997 |
| 3354.75 | 11.6172 |
| 3365.37 | 11.6281 |
| 3380.04 | 11.6431 |
| 3388.68 | 11.652 |
| 3402.72 | 11.6666 |
| 3421.98 | 11.6869 |
| 3431.88 | 11.6973 |
| 3453.66 | 11.7202 |
| 3464.64 | 11.7318 |
| 3475.08 | 11.7428 |
| 3495.42 | 11.7644 |
| 3508.38 | 11.7781 |
| 3541.77 | 11.8131 |
| 3564.36 | 11.8366 |
| 3601.62 | 11.8748 |
| 3609.72 | 11.8831 |
| 3630.96 | 11.9047 |
| 3658.95 | 11.9332 |
| 3671.28 | 11.9456 |
| 3682.89 | 11.9577 |
| 3704.58 | 11.9808 |
| 3725.55 | 12.003 |

S2.3: Linear equation linking the cumulative area of suitable parcels for willow production and weighed distance by the yield and area of each parcel for grassland type in central and northern New York. The area and transportation distance of each parcel to the end user were determined from a spatial analysis using GIS tools (from Yang, 2017).

Grassland

| Cumulative Area (ha) | Weighed distance (km) |
| --- | --- |
| 18 | 1.27491 |
| 27.72 | 2.26508 |
| 36.09 | 3.29816 |
| 47.43 | 4.14541 |
| 57.6 | 4.73639 |
| 70.29 | 5.6446 |
| 79.83 | 6.13953 |
| 90.63 | 6.67447 |
| 98.73 | 6.99999 |
| 108.27 | 7.5059 |
| 124.56 | 8.28032 |
| 133.65 | 8.64383 |
| 145.44 | 9.20208 |
| 158.4 | 9.73689 |
| 168.39 | 10.1771 |
| 189.18 | 11.0166 |
| 199.89 | 11.3916 |
| 207.99 | 11.6779 |
| 254.07 | 13.1118 |
| 262.17 | 13.3275 |
| 270.9 | 13.5468 |
| 282.42 | 13.824 |
| 292.32 | 14.0493 |
| 301.32 | 14.2465 |
| 322.56 | 14.6796 |
| 330.57 | 14.8354 |
| 345.69 | 15.1202 |
| 360.99 | 15.3883 |
| 385.11 | 15.7682 |
| 400.86 | 15.9968 |
| 418.86 | 16.2634 |
| 429.03 | 16.4054 |
| 438.66 | 16.5461 |
| 447.93 | 16.6765 |
| 467.73 | 16.9451 |
| 550.98 | 17.8763 |
| 569.07 | 18.0464 |
| 577.26 | 18.1242 |
| 600.3 | 18.3558 |
| 610.38 | 18.4519 |
| 622.98 | 18.5708 |
| 633.69 | 18.6686 |
| 648.72 | 18.8051 |
| 658.17 | 18.8911 |
| 669.87 | 18.9982 |
| 680.94 | 19.1001 |
| 694.71 | 19.2251 |
| 704.07 | 19.3127 |
| 713.07 | 19.3952 |
| 724.14 | 19.4937 |
| 751.32 | 19.7252 |
| 759.51 | 19.7928 |
| 772.47 | 19.9035 |
| 780.48 | 19.9769 |
| 791.19 | 20.0746 |
| 805.23 | 20.2013 |
| 815.04 | 20.2927 |
| 837 | 20.4942 |
| 849.42 | 20.6105 |
| 874.89 | 20.8684 |
| 890.46 | 21.0311 |
| 900.72 | 21.1364 |
| 910.35 | 21.2362 |
| 918.72 | 21.324 |
| 930.6 | 21.4468 |
| 951.57 | 21.663 |
| 978.03 | 21.9412 |
| 987.84 | 22.0414 |
| 1000.08 | 22.1681 |
| 1020.42 | 22.3817 |
| 1046.07 | 22.6458 |
| 1094.76 | 23.1145 |
| 1116.63 | 23.3155 |
| 1125.18 | 23.3926 |
| 1148.22 | 23.5953 |
| 1158.48 | 23.6842 |
| 1171.62 | 23.7977 |
| 1201.23 | 24.0445 |
| 1209.96 | 24.1152 |
| 1221.39 | 24.2077 |
| 1232.64 | 24.3011 |
| 1249.47 | 24.4462 |
| 1271.7 | 24.6342 |
| 1282.32 | 24.7219 |
| 1292.31 | 24.8043 |
| 1302.21 | 24.8852 |
| 1311.84 | 24.965 |
| 1332.9 | 25.1392 |
| 1345.59 | 25.2434 |
| 1390.59 | 25.598 |
| 1411.65 | 25.7581 |
| 1445.58 | 26.0063 |
| 1456.11 | 26.0848 |
| 1470.69 | 26.1933 |
| 1484.91 | 26.3035 |
| 1493.37 | 26.3698 |
| 1502.19 | 26.4393 |
| 1511.01 | 26.508 |
| 1520.82 | 26.5902 |
| 1537.65 | 26.7334 |
| 1545.66 | 26.8008 |
| 1553.67 | 26.8675 |
| 1562.22 | 26.9408 |
| 1577.34 | 27.0692 |
| 1589.94 | 27.1799 |
| 1599.21 | 27.2632 |
| 1609.2 | 27.3653 |
| 1619.19 | 27.4663 |
| 1627.83 | 27.5531 |
| 1640.07 | 27.6763 |
| 1652.04 | 27.7963 |
| 1661.76 | 27.8926 |
| 1676.79 | 28.0402 |
| 1691.82 | 28.1851 |
| 1700.19 | 28.2663 |
| 1713.15 | 28.3921 |
| 1755.63 | 28.8008 |
| 1767.69 | 28.9138 |
| 1776.6 | 28.9968 |
| 1794.78 | 29.1711 |
| 1803.42 | 29.2569 |
| 1812.06 | 29.3419 |
| 1847.61 | 29.6898 |
| 1856.43 | 29.7747 |
| 1865.34 | 29.8601 |
| 1874.34 | 29.946 |
| 1889.37 | 30.0898 |
| 1904.4 | 30.2314 |
| 1915.2 | 30.3328 |
| 1924.02 | 30.4158 |
| 1935.36 | 30.5224 |
| 1944.45 | 30.6072 |
| 1966.05 | 30.8073 |
| 1978.92 | 30.9284 |
| 1986.93 | 31.0037 |
| 1996.29 | 31.0912 |
| 2010.69 | 31.2243 |
| 2025.09 | 31.3555 |
| 2037.96 | 31.4718 |
| 2048.31 | 31.5656 |
| 2057.04 | 31.6442 |
| 2065.77 | 31.7222 |
| 2075.67 | 31.8099 |
| 2085.57 | 31.8968 |
| 2093.67 | 31.969 |
| 2105.55 | 32.0744 |
| 2116.62 | 32.1721 |
| 2127.69 | 32.2688 |
| 2146.5 | 32.4313 |
| 2167.56 | 32.6112 |
| 2175.84 | 32.682 |
| 2194.92 | 32.8448 |
| 2205.45 | 32.9353 |
| 2215.98 | 33.0249 |
| 2224.98 | 33.1021 |
| 2233.98 | 33.1788 |
| 2248.92 | 33.3079 |
| 2265.84 | 33.4566 |
| 2281.41 | 33.5918 |
| 2294.91 | 33.7098 |
| 2303.82 | 33.7876 |
| 2311.83 | 33.8575 |
| 2319.84 | 33.927 |
| 2335.5 | 34.0637 |
| 2348.19 | 34.1737 |
| 2361.24 | 34.286 |
| 2372.4 | 34.3811 |
| 2383.56 | 34.4758 |
| 2395.89 | 34.5797 |
| 2406.69 | 34.6704 |
| 2418.21 | 34.7664 |
| 2430.72 | 34.8697 |
| 2443.23 | 34.9719 |
| 2453.76 | 35.0577 |
| 2465.1 | 35.1501 |
| 2476.44 | 35.2417 |
| 2487.06 | 35.3269 |
| 2496.15 | 35.4001 |
| 2507.4 | 35.4901 |
| 2527.47 | 35.6491 |
| 2547.54 | 35.8055 |
| 2557.62 | 35.8833 |
| 2566.53 | 35.9518 |
| 2575.26 | 36.0195 |
| 2586.24 | 36.1076 |
| 2597.22 | 36.195 |
| 2608.29 | 36.2834 |
| 2618.46 | 36.3641 |
| 2626.65 | 36.4297 |
| 2634.84 | 36.4949 |
| 2646.63 | 36.5883 |
| 2661.21 | 36.7058 |
| 2677.32 | 36.8361 |
| 2693.43 | 36.9649 |
| 2704.23 | 37.0506 |
| 2712.78 | 37.1181 |
| 2731.32 | 37.264 |
| 2741.22 | 37.3426 |
| 2751.84 | 37.4265 |
| 2761.2 | 37.4999 |

Yang, S. Understanding Land Use and Land Cover Change: National Forest Dynamics and Regional Willow Crop Environmental Impacts Based on Spatial Modeling and Spatial Life Cycle Assessment. (SUNY College of Environmental Science and Forestry, 2017).
